# SPEAR UltraDetect™ pTau‐217: A High‐Accuracy, Scalable Plasma Biomarker for PET Confirmed Cognitively Impaired Patients Using a Single Cutoff

**DOI:** 10.1002/alz70856_106293

**Published:** 2026-01-12

**Authors:** Tsz Wing Fan, WonHee Kim, Corinne Thomas, Jeremy Tan, Wenjing Jiang, Feng Xuan

**Affiliations:** ^1^ SPEAR BIO INC, Woburn, MA, USA

## Abstract

**Background:**

Alzheimer's disease misdiagnosis in specialized clinics and primary care often delays confirmatory testing and treatment windows for anti‐amyloid therapies. Plasma pTau‐217 assays offer a minimally invasive, scalable alternative for detecting amyloid pathology, yet their cost‐effectiveness in reducing confirmatory testing remains uncertain. Many current assays fail to surpass 90% sensitivity and specificity with a single cutoff, relying instead on a tiered double‐cutoff approach that inflates accuracy by discounting the intermediate zone (>20%). Stricter thresholds further expand this gray zone, leaving a substantial number of patients with delayed treatment decisions. To improve diagnostic accuracy, normalization strategies incorporating non‐phosphorylated tau or amyloid‐beta ratios were explored to mitigate false positives from commodities like CKD beyond pTau‐217 alone.

**Method:**

We present the SPEAR UltraDetect™ pTau‐217 immunoassay, which employs a unique two‐factor authentication mechanism and a homogenous assay format, allowing free analyte‐binder interaction for maximized specificity. The test is semi‐automated (20 min hands‐on time) and achieves >90% clinical accuracy for amyloid pathology using a single cutoff. The assay requires only 1 µL of diluted plasma per measurement and operates on a wash‐free workflow with qPCR readout. A GAP (Global Alzheimer's Platform) cohort diagnosed with MCI (*n* = 67) or mild dementia (*n* = 34) with amyloid PET‐confirmed status (44 negative, 57 positive) was evaluated. Assay performance was compared to the MSD assay with Eli Lilly antibody, assessing specificity and fold‐change using single and double‐cutoff strategies.

**Result:**

The SPEAR pTau‐217 assay demonstrated a 4.9‐fold increase in pTau‐217 levels in PET‐positive cases, nearly doubling the 2.5‐fold seen with the MSD assay with Eli Lilly antibody. Using a single cutoff, SPEAR achieved 91.2% sensitivity, 93.2% specificity, 94.6% PPV, and 89.1% NPV, with 92.1% overall accuracy. When optimizing for ≥95% specificity and sensitivity, the indeterminate range was reduced from 24.8% (MSD Eli Lilly antibody) to 13.9%. The assay exhibited robust inter‐day precision (5% CV) and 99% concordance across runs.

**Conclusion:**

The SPEAR UltraDetect™ pTau‐217 assay offers improved clinical differentiation of amyloid PET status with > 90% accuracy using a single cutoff. Its minimal sample requirement, wash‐free workflow, and qPCR compatibility make it potentially a scalable, cost‐effective test for determining amyloid pathology of AD.

